# Locus-Specific Mutation Databases for Neurodegenerative Brain Diseases

**DOI:** 10.1002/humu.22117

**Published:** 2012-05-11

**Authors:** Marc Cruts, Jessie Theuns, Christine Van Broeckhoven

**Affiliations:** 1 Neurodegenerative Brain Diseases Group, Department of Molecular Genetics, VIBAntwerpen, Belgium; 2 Laboratory of Neurogenetics, Institute Born-Bunge, University of AntwerpAntwerpen, Belgium

**Keywords:** locus-specific, mutation database, neurodegenerative brain disease, Alzheimer disease, frontotemporal lobar degeneration, Parkinson disease

## Abstract

The Alzheimer disease and frontotemporal dementia (AD&FTLD) and Parkinson disease (PD) Mutation Databases make available curated information of sequence variations in genes causing Mendelian forms of the most common neurodegenerative brain disease AD, frontotemporal lobar degeneration (FTLD), and PD. They are established resources for clinical geneticists, neurologists, and researchers in need of comprehensive, referenced genetic, epidemiologic, clinical, neuropathological, and/or cell biological information of specific gene mutations in these diseases. In addition, the aggregate analysis of all information available in the databases provides unique opportunities to extract mutation characteristics and genotype–phenotype correlations, which would be otherwise unnoticed and unexplored. Such analyses revealed that 61.4% of mutations are private to one single family, while only 5.7% of mutations occur in 10 or more families. The five mutations with most frequent independent observations occur in 21% of AD, 43% of FTLD, and 48% of PD families recorded in the Mutation Databases, respectively. Although these figures are inevitably biased by a publishing policy favoring novel mutations, they probably also reflect the occurrence of multiple rare and few relatively common mutations in the inherited forms of these diseases. Finally, with the exception of the PD genes *PARK2* and *PINK1*, all other genes are associated with more than one clinical diagnosis or characteristics thereof. Hum Mutat 33:1340–1344, 2012. © 2012 Wiley Periodicals, Inc.

## Introduction

Neurodegenerative brain diseases are adult-onset diseases in which degeneration of specific neuronal populations of the central nervous system plays a central role. The most common neurodegenerative brain diseases are Alzheimer disease (AD; MIM# 104300), Parkinson disease (PD; MIM# 168600), and frontotemporal lobar degeneration (FTLD; MIM# 600274) and their prevalence is steadily increasing in the absence of effective therapies. AD, PD, and FTLD are proteinopathies in which the toxic aggregation and deposition of characteristic proteins in specific brain areas are major etiologic and diagnostic hallmarks [Yankner et al., 2008]. Genetics plays a major role in all three diseases, which in general result from a complex combination of multiple genetic risk and protective factors, in concert with environmental factors constituting an individual's risk to develop the disease at a given point in life. However, AD, FTLD, as well as PD have an infrequent monogenic component in which a highly penetrant Mendelian inherited dominant or recessive mutation invariantly leads to disease, be it often at variable and largely unpredictable ages.

Knowledge of monogenic mutations leading to neurodegenerative brain diseases is of great value for several reasons. In clinical genetic counseling, for example, knowledge of pathological mutations and their genic location will assist in working out efficient diagnostic screening protocols. Moreover, when mutation screening is used to support or specify a clinical diagnosis, a quick survey of parameters such as evidence of familial cosegregation, frequencies of occurrence in patients and unaffected individuals, interspecies codon conservation, cell biological consequences, and genotype–phenotype correlations might assist in making an accurate diagnostic decision prior to treatment. In a research setting, knowledge of the type and location of multiple pathological mutations in a specific disease gene might reveal valuable indications towards functionally critical protein domains and/or motifs and disease mechanisms.

The AD&FTLD and PD Mutation Databases described in this manuscript aim to provide this information for the most commonly mutated genes in a comprehensive way. The AD&FTLD Mutation Database is a locus-specific database (LSDB) that was conceived in 1998 [Cruts and Van Broeckhoven, 1998b] in the perspective of the Mutation Database Initiative [Cotton et al., 1998], an initiative originally fostered by the Human Genome Organization (www.hugo-international.org) that has through the years evolved to the Human Genome Variation Society (HGVS, www.hgvs.org). HGVS is dedicated to promoting and supporting of collection, documentation, and free distribution of genomic variation information and associated clinical variations. From the start, the AD&FTLD Mutation Database stores curated genetic, clinical, and biological information of DNA variations in the Mendelian AD genes *APP*, *PSEN1*, and *PSEN2* (Table 1) [Cruts and Van Broeckhoven, 1998a]. Because of observed genetic overlaps between the etiology of both, AD and FTLD, all known Mendelian FTLD genes (Table 1) were added to the AD&FTLD Mutation Database from 2004 onward [Gijselinck et al., 2008; Rademakers et al., 2004]. The PD Mutation Database was set up in 2010 [Nuytemans et al., 2010], essentially in response to the lack of comprehensive LSDBs of PD genes. Today, it contains extended genetic and clinical information of variations in the five most common Mendelian PD genes (Table 1). The primary user interfaces of the databases are publicly accessible dedicated websites: www.molgen.ua.ac.be/ADMutations and www.molgen.ua.ac.be/FTDMutations for the AD&FTLD Mutation Database, and www.molgen.ua.ac.be/PDmutDB for the PD mutations database. In addition, basic genetic information of the mutations is shared with the Gen2Phen project [Webb et al., 2011] and NCBI's dbSNP [Sayers et al., 2012].

**Table 1:** Genes Catalogued in the AD&FTLD and PD Mutation Databases

Primary disease	Gene	Genomic location	Mutations (clinical/unclear/ benign coding)	Unrelated individuals or families	References^a^
AD	*APP*	21q21.2	32/1/6	90	86
	*PSEN1*	14q24.3	185/8/4	411	217
	*PSEN2*	1q42.13	13/7/5	34	39
FTLD	*C9orf72*	9p21.2	1/0/0	336	5
	*CHMP2B*	3p11.2	4/4/4	12	11
	*FUS*	16p11.2	23/4/18	54	15
	*GRN*	17q21.32	69/35/45	264	89
	*MAPT*	17q21.1	44/2/27	138	172
	*TARDBP*	1p36.22	34/2/9	95	26
	*VCP*	9p13.3	17/2/0	45	26
PD	*LRRK2*	12q12	6/68/54	1,051	176
	*PARK2*	6q26	127/65/22	777	164
	*PARK7*	1p36.23	6/17/5	31	26
	*PINK1*	1p36	28/80/30	190	69
	*SNCA*	4q22.1	25/1/1	51	49

Total	14		614/296/230	3,579	1,127

a Journal article or personal communication.

## Database Content

### Variation Inclusion Policy

The AD&FTLD and PD Mutation Databases aim to provide an up to date catalogue of all gene variations linked to disease causation or predicted to affect the encoded protein sequence. Because the knowledge of benign coding variations is as important as the knowledge of clinical variations, these are also documented. Noncoding neutral variations are excluded because in most genes they outnumber coding variations and (1) are not the main interest of the database user, (2) locate in regions that are not included in standard exon-based mutation screening strategies, and (3) are rarely documented in detail in literature reports, the major resource of the database content. As a result, comprehensively cataloging these variations is not realistic in the context of the LSDBs and competing against other variation databases such as NCBI's dbSNP, that are much better placed to catalog large numbers of this type of variations, makes little sense. For example, dbSNP build 135 holds 3,654 common variations in the human FTLD-associated gene *MAPT* [Cruts et al., 2005]. Most of these variations are located deeply in introns, while the 43 known clinical mutations are in coding regions of exons 1, and 9–13, and the first 19 nucleotides of intron 10 [Rademakers et al., 2004]. Information of common variations outside these genic regions is not of direct interest to the clinical and molecular geneticists consulting the AD&FTLD Mutation Database. Obviously, common variations associated with disease susceptibility are stored in separate, dedicated databases specialized to hold other descriptive parameters [Lill and Bertram, 2012]), for example, AlzGene [Bertram et al., 2007], and PDGene [Lill et al., 2012].

Genes currently included in the AD&FTLD and PD Mutation Databases are shown in Table 1. Repeat expansion mutations pose specific database issues [Martindale et al., 2012] and all pathological expansions of the noncoding G_4_C_2_ repeat in the *C9orf72* promoter leading to FTLD and amyotrophic lateral sclerosis (ALS) are considered the same mutation. The PD Mutation Database contains gene variations of the five genes segregating the majority of PD mutations (Table 1).

Next to personal communications (<1%), scientific literature (>99%) is the major data source. The NCBI PubMed literature database is periodically scanned using as query the genes' official symbols and full names as designated by the Human Gene Nomenclature Committee [Seal et al., 2011], complemented with all gene aliases listed in the NCBI Gene database or commonly used in literature. Retrieved publications are scanned for the presence of information on gene variations. Variation names are checked for consistency with the current HGVS guidelines for description of sequence variations [den Dunnen and Antonarakis, 2000] (www.hgvs.org/mutnomen). Because ambiguous variation names are not uncommon in literature, reliability criteria are employed to evaluate the genuineness of the described variation. Variations consistently described following two or more naming systems, for example, transcript and protein, are considered reliable. In addition, the publication is searched for other evidence, for example, a DNA sequence fragment specifying the variations. When the nature of the variation remains ambiguous, the authors are contacted. To maintain database content integrity, variations that remain ambiguous are excluded.

### Genetic and Clinical Documentation

Variations are stored in the databases with names according to the HGVS guidelines, but commonly used aliases are also given. Variant description is shown at the level of gene, transcript, and protein, indicating the affected region and whether the variation is experimentally observed in at least one report or predicted based on indirect evidence, for example, a predicted protein variation based on a transcript sequence and codon translation table. Variation position numbering is relative to stable RefSeq reference sequences [Pruitt et al., 2009] for gene (RefSeqGene), RNA, and protein. For historical reasons, genomic numbering is also given relative to non-RefSeqGene sequences for some genes. Mutalyzer [Wildeman et al., 2008] is used to assist in generating or verifying variation names. All details are documented with a comprehensive list of literature references.

In the PD Mutation Database, genetic information, primary clinical diagnosis, ages at onset and death, and disease duration are stored for each individual documented in literature. Genetic test results and neuropathological confirmation of the diagnosis are also recorded for each documented family member. Availability of these details of each individual within pedigrees is useful to form an opinion of cosegregation and penetrance of a variation with respect to zygosity. In contrast to the PD Mutation Database, in the AD&FTLD Mutation Database, all information is stored at the level of the family, including family averages and ranges of clinical parameters such as ages at onset and death.

Together, the wealth of information stored in the AD&FTLD and PD Mutation Databases have revealed interesting observations. Looking at the number of occurrences of each variation, it is striking that as much as 60–64% of clinical variations are private to one single family in AD, FTLD, as well as PD, while only 3–8% of mutations occur in 10 or more families. It should be noted that literature is inevitably biased toward novel mutations because publishing known mutations is hindered by the limited degree of novelty and underestimated informative value. Moreover, an unknown number of mutations identified in molecular diagnostic environments where publishing is not a priority never reaches the public domain. These biases are reflected in the data available in the Mutation Databases and consequently, private mutations are most probably overestimated. However, at least these data suggest that the genetic basis of Mendelian forms of AD, PD, and FTLD consist primarily of multiple rare mutations in the known disease genes. Consequently, mutation screening strategies aiming to detect only known mutations have limited value. Oppositely few relatively common mutations do exist: the five mutations with the highest number of independent observations for each disease are reported in 21% of AD, 43% of FTLD, and 48% of PD families recorded in the Mutation Databases. The high proportion in PD is explained by the *LRRK2* p.Gly2019Ser mutation, which in itself explains 34% of all PD families. The high relative occurrence of some variations is explained by a founder effect in specific geographic regions and frequencies might differ considerably in other regions. Recorded geographic origin and ethnicity of the mutations carriers allow to map the geographical distribution of specific variations and the identification of population-specific founder mutations. On the basis of this information, it is advised to work out region-specific gene and exon screening priorities.

**Figure 1:**
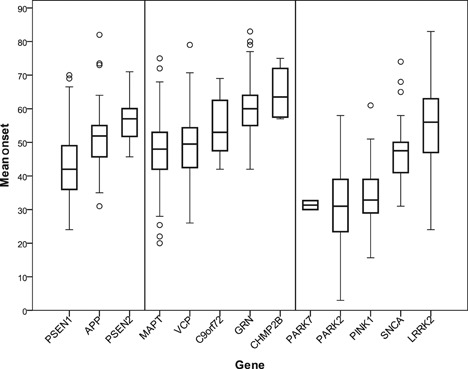
Boxplot showing disease onset age distributions per gene. Family-based average onset ages of established pathogenic variants were used. For *PARK7*, *PARK2*, and *PINK1*, only patients carrying homozygous or compound heterozygous mutations were included in the calculations. Boxes represent the interquartile onset age distribution, horizontal lines indicate medians, whiskers show standard deviations, and circles indicate outliers.

It is apparent that all AD, FTLD, and PD genes are associated with a wide-onset age range, although Mendelian mutations are on average associated with a disease onset before the age of 65 years (Fig. 1). *PARK7* mutations are associated with onset ages of PD in the narrow range between 30 and 33 years; however, this is based on two families only, in which patients carry a homozygous mutation with established pathogenicity. In AD, earliest disease onset ages are recorded in *PSEN1* mutation carriers, in whom the disease starts on average 8.4 years earlier than in *APP* mutation carriers (average 42.9 vs. 51.3 years) and 14.2 years earlier than in *PSEN2* mutation carriers (average 57.1 years) (Fig. 1). In FTLD, the earliest average onset age is associated with *MAPT* (47.9 years) and *VCP* (49.5 years) mutations. Intermediate onset ages of an average 55 years are noted in FTLD patients with a *C9orf72* expanded hexanucleotide repeat. *GRN* mutations are associated with an average onset age of 59.3 years, and *CHMP2B* mutations with 64.8 years. Homozygous or compound heterozygous *PARK2* and *PARK7* may cause juvenile onset of PD with average onset ages of 31.2 and 31.3 years, respectively. Onset age in *SNCA* mutation carriers is on average 15.6 years later (average 46.9 years), while *LRRK2* mutation carriers have on average the latest onset age (55.2 years) (Fig. 1). Importantly, in all three diseases, even interquartile onset age ranges overlap substantially among all genes, meaning that onset age is not an absolute discriminator of mutant gene (Fig. 1).

When considering the correlation between primary clinical diagnoses and mutant gene, each gene is strongly associated with one clinical diagnosis (Table 2). However, genetic overlaps between the different neurodegenerative brain diseases and the clinical scope of each gene are significant. *PSEN1* and *2*, *MAPT*, and *GRN* mutations are associated with clinical characteristics typical of AD, FTLD, and/or PD in a substantial number of patients (Table 2). Strikingly, all genes have been associated with clinical characteristics of PD, with the exception of FTLD genes *C9ORF72, VCP*, and *CHMP2B* (Table 2). These latter genes however were in addition to FTLD also associated with ALS, inclusion body myopathy, and Paget disease. *PINK1* and *PARK2* mutations appear specific of a clinical diagnosis of PD; however, *PARK2* mutations have also been associated with dopa-responsive dystonia [Clot et al., 2009].

**Table 2:** Clinical Presentation Associated with Each Disease Gene

		Gene												
		
		PD					AD			FTLD				
				
		*LRRK2*	*PINK1*	*PARK2*	*SNCA*	*PARK7*	*APP*	*PSEN1*	*PSEN2*	*MAPT*	*GRN*	*VCP*	*CHMP2B*	*C9orf72*
Primary diagnosis	PD	>99%	100%	>99%	100%	89%	1%	1%	5%	8%	1%			
	AD	<1%			10%	11%	100%	99%	95%	8%	4%			
	FTLD	<1%						2%	14%	97%	97%	93%	60%	29%

Shown are percentages of independent observations of mutations in a given gene that are associated with clinical characteristics that are typical of the respective primary diagnosis. Primary diagnoses other than PD, AD, or FTLD (e.g., ALS) are not shown but were included in the calculations.

Taken together, the AD&FTLD and PD Mutation Databases are useful tools to work out a gene and exon priority scheme for mutation screening, for example, in the context of clinical genetic counseling. As illustrated above, parameters to be taken into account are clinical diagnosis, onset age, family history, and regional mutation frequencies. Importantly, although the Mendelian disease variations are generally associated with familial patients with early onset of disease, a mutation cannot formally be excluded from patients with a late-onset age above 65 years, or patients without noted family history. Obviously, in the total size of this group of patients, the relative occurrence of Mendelian mutations is low due to the high number of patients with a complex non-Mendelian disease etiology.

### Effect on Protein Function

Consequences of the mutation on gene function are included in the Mutation Databases as far as their relevance to the disease mechanism is demonstrated. For example, in AD, a well-established disease mechanism is the amyloid β cascade [Hardy, 2006], which states that the relative increase of Aβ_42_ production is at the basis of AD biology. Mutations in the Aβ precursor APP or the proteases PSEN1 and PSEN2 play established roles in Aβ production [Hardy, 1997]. Therefore, in vivo and in vitro evidence of the effect of AD mutations on Aβ production is recorded in the AD&FTLD Mutation Database [Theuns et al., 2006]. Similarly, established biological consequences of *MAPT* mutations are their effect on expression of 4R/3R tau protein ratio, microtubule assembly, and tau filament formation [Rademakers et al., 2004] and experimental data in this context are also included in the AD&FTLD Mutation Database.

**Figure 2:**
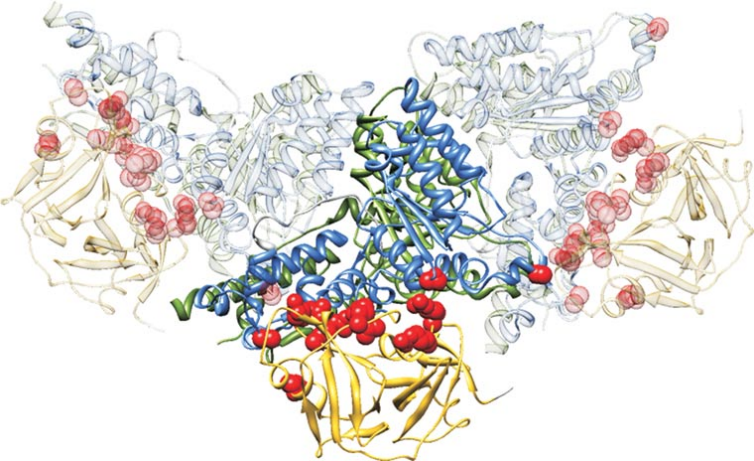
Example of graphical presentation of gene mutations. Shown is the example of VCP mutations in the 3D organization of three monomers of the homohexameric protein organization (PDB entry 3CF3). Mutations are shown as red atoms on the ribbon presentation of the CDC48-like N-terminal domain (yellow), and D1 (blue) and D2 (green) ATPase domains, clearly demonstrating the alignment of mutations at the interface between the CDC48 and D1 domains [Weihl et al., 2009].

When the disease mechanism is not well known or the role of genes and mutations in the biology of disease is not established, the location of the variation in the protein and its predicted effect on protein function are useful indicators. Therefore, two-dimensional (2D) and/or three-dimensional (3D) graphic protein presentations showing the variation position with respect to protein domain are provided in the Mutation Databases. For example, all known pathogenic *APP* mutations are located at one of three secretase sites or inside the Aβ peptide sequence and affect Aβ production or aggregation properties, respectively [Brouwers et al., 2008], which can be appreciated from the provided 2D protein mutation map. Similarly, all known pathogenic *VCP* mutations are located at the interface between the N-terminal CDC48-like domain and the D1 ATPase domain [Weihl et al., 2009], resulting in reduced affinity for ADP [Tang et al., 2010]. Therefore, even though the exact role of VCP mutations in disease biology is unknown, the amino acid position is useful to predict pathogenecity and can only be appreciated from the 3D structural map provided in the AD&FTLD Mutation Database (Fig. 2).

For similar reasons, the Mutation Databases also provide the option to show an overview of the mutations as a custom track in the genome browser of the University of California Santa Cruz (UCSC), offering the benefit to interpret the location of mutations relative to the host of available genomic annotation tracks, like amino acid conservation for coding variations and regulatory potential for noncoding variations.

## Future Prospects

The AD&FTLD and PD Mutation Databases are established resources for clinical geneticists, neurologists, and researchers alike receiving a steadily increasing number of independent visits each year, exceeding 27,000 visits in 2011. Major efforts are being done to keep the content of the databases up to date and direct submissions are encouraged to also include unpublished variants. Database inclusion of the more recently identified PD genes *ATP13A2*, *EIF4G1*, *FBXO7*, *GBA*, *GIGYF2*, *HTRA2, VPS35*, and *UCHL1* is planned.

The AD&FTLD Mutation Database was designed to hold dominant, heterozygous mutations only, preventing the allocation of multiple variations to one family, for example, recessive mutations such as the homozygous *APP* p.Ala673Val mutation affecting Aβ amyloidogenesis [Di Fede et al., 2009]. It is expected that more such mutations will be identified with the on-going scaling-up of gene sequencing efforts to analyze extended gene sets in larger, clinically less strictly defined patient series. A major database improvement will be the merging of the AD&FTLD and the PD Mutation Databases, facilitating the storage of recessive mutations for AD and FTLD as is already the case for PD. Also with the upcoming naturalization of next-generation sequencing technologies, multigenic causes of disease will probably be revealed and the structure of Mutation Databases is being prepared for that. Importantly, this unified mutation database for the major neurodegenerative brain diseases will better accommodate the clinicogenetic overlaps between PD, AD, and FTLD. Also, in this respect, further improvement of the implementation of clinical data and genotype–phenotype correlates will be established by the implementation of phenotypic ontology [Köhler et al., 2012], allowing to associate genetic variations with specific clinical characteristics rather than disease diagnoses.

Finally, defining pathogenic nature of a variation is not a trivial issue and specifying general criteria is a matter of much debate. For mutations in the AD genes *APP*, *PSEN1*, and *PSEN2*, an algorithm has been proposed, primarily based on segregation information and effect on Aβ processing [Guerreiro et al., 2010]. In more general terms, conclusive evidence is problematic in the absence of solid familial segregation evidence, especially when established in vitro or in vivo biochemical readouts are unavailable. Especially in the case of recessive disease genes, segregation evidence is rarely obtained in adult-onset diseases. Deployment of a transparent probability estimation system of the pathogenic nature of a variation based on parameters including segregation, absence in individuals a neurodegenerative disorder, location within the protein, interspecies amino acid conservation, in vitro and in vivo evidence and predicted variation characteristics is being developed.
